# Ovarian antral follicle populations and embryo production in cattle

**DOI:** 10.21451/1984-3143-AR2018-0072

**Published:** 2018-08-17

**Authors:** Amanda Fonseca Zangirolamo, Fabio Morotti, Nathalia Covre da Silva, Tamires Korchovei Sanches, Marcelo M. Seneda

**Affiliations:** 1 Universidade Estadual de Londrina, Laboratório de Reprodução Animal (DCV-CCA-UEL), Londrina, PR, Brazil.; 2 National Institute of Science and Technology for Dairy Production Chain (INCT–LEITE), Universidade Estadual de Londrina, Londrina, PR, Brazil.

**Keywords:** antral follicle count, *Bos taurus*, *Bos indicus*, cow, fertility

## Abstract

Reproductive biotechniques such as embryo production are important tools to increase the reproductive performance in cattle in a short time. In this context, the antral follicle count (AFC), which reflects the population of antral follicles present in an ovary, has been indicated as an important phenotypic characteristic related to female fertility and closely correlated to the performance of *in vivo* and *in vitro* embryo production (IVEP). A positive correlation was evidenced between AFC and oocyte retrieval by ovum pick up (OPU) sessions and and with the number of embryos produced. Several studies have reported that females with a high AFC had greater embryo yields compared to those with medium and low AFC. However, controversial results were obtained by studies conducted in different bovine breeds. Many conflicting data may be due to the differences in the experimental design, particularly regarding the classification of animals in AFC groups, subspecies particularities, herd aptitude or even issues related to animal management. Therefore, aspects such as the choice of donor, type of aspirated follicles and the stage of follicular wave need to be clarified. Thus, this text aims to discuss the use of AFC as a reproductive tool and its applications in the *in vivo* and *in vitro* production of embryos, besides describing consistent results and new challenges regarding AFC and embryo production.

## Introduction

In the world scenario for bovine embryos, in the last decade, the *in vitro* embryo production (IVEP) has expanded remarkably when compared to *in vivo* embryo production ( Watanabe *et al*., 2017 ). In the year 2016, a total of 666,215 *in vitro* embryos were produced, exceeding for the first time the volume of embryos produced *in vivo* ( Perry, 2017 ). In this context, Brazil has contributed to the consolidation of large-scale application of this biotechnique and many challenges have been faced to improve the IVEP ( Perry, 2016 ).

Several studies have been conducted to maximize the reproductive efficiency of the herd. In this way, the ovarian antral follicular count (AFC) as a tool to evaluate the ovarian reserve, has been positively correlated with parameters such as the number of viable oocytes, blastocyst ( Santos *et al*., 2016 ) and conception rate after AI ( Mossa *et al*., 2012 ). Different research groups investigated the embryo production performance of females with different AFC. This parameter is variable among females but highly constant in the same female ( Burns *et al*., 2005 ; Morotti *et al*., 2017 ). For both *indicus* and *taurus* subspecies and *indicus* x *taurus* crossbred, a positive correlation was found between AFC and oocyte retrieval in the ovum pick up (OPU) sessions and the number of embryos produced *in vitro* ( Ireland *et al*., 2008 ; Monteiro *et al*., 2017 ).

Considering embryonic production, females with high AFC presented a larger number of *in vitro* embryos when compared to those with low AFC ( Ireland *et al*., 2008 ; Monteiro *et al*., 2017 ). Such information is quite predictable, considering that more follicles provide more oocytes to the *in vitro* embryo production. On other hand, the influence of AFC on the efficiency of embryonic production remains not well understood. For example, Rosa *et al*. (2018) , reported no differences in both cleavage and blastocyst rates of oocytes that came from ovaries with different AFC. Also evaluating different AFC patterns, Rosa *et al*. (2018) evaluated the genes involved in oogenesis and folliculogenesis, which were differentially expressed in granulosa (*progesterone receptor - PGR* and *Anti-müllerian hormone receptor type II - AMHR2*) and cumulus cells (*natriuretic peptide receptor 2 and 3 - NPR2/ NPR3, fibroblast growth factor 10 - FGF10* and *signal transducer and activator of transcription 3 - STAT3*) from high versus low AFC cows. Mossa *et al*. (2010) reported lower abundance of *cytochrome P450 family 17 subfamily A member 1 - CYP17A1* mRNA in thecal cells of low versus high AFC. Moreover, the study by Ireland *et al*. (2009) reported that the abundance of mRNAs for *cytochrome P450 family 19 subfamily A member 1 - CYP19A1* in granulosal cells and *estrogen receptor 1 and 2 - ESR1/ ESR2* , and *cathepsin B - CTSB* in cumulus cells were greater, whereas mRNAs for AMH in granulosal cells and *TBC1 domain family member 1 - TBC1D1* in thecal cells were lower for animals with low compared to the high AFC group during follicle waves.

Considering a general view on AFC, many points need to be addressed. For example, there is no standard to the classification of AFC according to the number of follicles. Comparing all the reports at the literature, it is easy to recognize several patters of AFC groups. In this way, it is quite difficult to compare data among the articles. Therefore, considering the importance of AFC on the embryo production this text aims to discuss: i) The use of AFC as a reproductive tool and the relationship between different fertility parameters (i.e. blastocyst rate and pregnancy, AMH concentration, among others); ii) applications of AFC on the *in vivo* and *in vitro* production of embryos and, iii) controversial data and new challenges regarding to the practical use of AFC as a reproductive tool for cattle.

## Use of AFC as a reproductive tool

The association between reproductive biotechniques and genetic improvement programs in cattle has contributed to the development of the milk and food production ( Hansen, 2014 ). The selection of females suitable for breeding is of great importance for reproductive efficiency, mainly because some ovarian physiological characteristics can directly influence the number and quality of oocytes ( Lonergan *et al*., 1994 ). In this context, cows can be classified as low, intermediate or high AFC, according to the number of antral follicles (of 3 mm in diameter) detected via ovarian ultrasonography.

The AFC has been related to fertility parameters such as ovarian size, corpus luteum diameter, number of healthy oocytes, endometrial thickness, progesterone concentration, pregnancy rate and calving interval ( Jimenez-Krassel *et al*., 2009 , Martinez *et al*., 2016 ). Thus, considering the relationship between AFC and several characteristics linked to fertility, the selection of donors could be performed using a single ultrasound examination at the beginning of reproductive life ( Silva-Santos *et al*., 2014 ; Morotti *et al*., 2017 ). Also, the Anti-mullerian hormone (AMH), has been recognized as an indicator of ovarian response to superovulation protocols ( Souza *et al*., 2015 ), being AMH also highly correlated to the AFC and viability of the oocytes ( Baruselli *et al*., 2016 ). It was also observed a positive association between AMH and fertility in dairy cows ( Jimenez-Krassel *et al*., 2015 ).

The AFC is a characteristic of low to moderate heritability, and it was verified that there is no correlation between AFC and milk production during lactation in Holstein cows ( Walsh *et al*., 2014 ). Similarly, for Braford (beef) cattle, some parameters related to meat production showed low correlation with AFC ( Morotti *et al*., 2017 ). Then, based on these studies, it can be suggested that AFC selection does not cause genetic demerit for the progeny.

In general, a greater number of antral follicles results in better OPU/IVEP quantitative efficiency. Many reports suggest that AFC is positively associated with the number of embryos produced by the donor in different breeds ( Ireland *et al*., 2008 ; Silva-Santos *et al*., 2014 , Santos *et al*., 2016 ). Although, more recently, Monteiro *et al*. (2017) did not find any advantage for *Bos indicus* females with high AFC relative to IVEP rates (Blastocyst rate 33.9% and 34.2% for low and high AFC animals, respectively).

In taurine females submitted to fixed time artificial insemination (FTAI) presenting high AFC, it was also found a higher pregnancy rate when compared to those with low AFC (94% and 84%, respectively; Mossa *et al*., 2012 ). In contrast, studies with indicine cattle did not find a positive correlation between AFC and pregnancy rates with artificial insemination ( Mendonça *et al*., 2013 ; Santos *et al*., 2016 ). Surprisingly, an evaluation of AFC and timed artificial insemination showed better reproductive performance in low AFC Nelore cows than high AFC females (conception rate 61.7% and 49.5%, respectively; Morotti *et al*., 2018 ). Recently, another unexpected result came from New Zealand with *Bos taurus* dairy cattle. The results showed that females with high AFC (≥ 25 follicles) had suboptimal fertility and shorter productive life when compared to those with low AFC (≤ 15 follicles) ( Jimenez-Krassel *et al*., 2017 ). In contrast, also working with dairy cattle, Modina *et al*. (2014) had reported that those cows with intermediate number of antral follicles (n < 10) were identified with reduction in the fertility parameters, when compared to females with > 10 follicles. Once again, the comparison between the articles is quite difficult considering that AFC was not the single condition that was different in the experimental design. As pointed by Martinez *et al*. (2016) , the associations of AFC with other fertility parameters need for further evaluations to ensure the best way to use this strategy on reproductive programs.

In conclusion, the relationship between AFC and fertility needs to be clarified. Both in *Bos taurus* and *Bos indicus* cattle have shown conflicting data, mainly when different approaches, embryo transfer - ET or artificial insemination – AI, have been considered.

## Applications of AFC for *in vitro* and *in vivo* production of embryos

The embryo yield is highly variable in both *in vitro* and *in vivo* embryo production systems ( Pontes *et al*., 2010 ). The IVP may be affected by oocyte recovery rate on OPU, as well as ET is affected by the response of the donor afterthe superovulation protocol (SOV) - ( Ireland *et al*., 2007 ; Silva-Santos *et al*., 2014 ). Therefore, it is interesting to consider that the success of these techniques is dependent on the individual ovarian characteristics of the donor, which may affect the number and quality of the oocytes / embryos that are recovered ( Stojsin-Carter *et al*., 2016 ).

In 2007, Ireland and collaborators performed a study with *Bos taurus* cattle and observed that the number of IVF embryos per animal in the high AFC group (≥ 25 follicles) was four times higher (P < 0.05) than the low AFC group (≤ 15 follicles). In the multiple ovulation and embryo transfer (MOET), the number of embryos recovered after artificial insemination was 10.6 ± 2.7 *vs.* 4.7 ± 0.7 for the high and low AFC group, respectively (P < 0.05).

Similarly, *indicus-taurus* donors with high AFC (≥ 40 follicles), produced a higher (P < 0.05) number of blastocysts than animals of low AFC group (≤ 10 follicles) both in IVF and MOET approaches ( Silva-Santos *et al*., 2014 ). However, the same team reported less consistent results for *Bos indicus* cattle. The high AFC showed better embryo production performance, but the cleavage rate was similar among low (≤ 7 follicles), intermediate (18 to 25 follicles) and high AFC (≥ 40 follicles) ( Santos *et al*., 2016 ).

The relationship between of the ovarian pool of follicles and the response to the superovulation has been previously evaluated ( Cushman *et al*., 1999 ). However, possible applications of AFC and specific protocols to SOV is topic that remains to be better understood.

Considering AFC and genes related to folliculogenesis and oogenesis, it was reported differences in gene expression in cumulus and granulosa cells collected from Nelore cows with low and high AFC. Interestingly, in the study by Rosa *et al*. (2018) , animals com high AFC had genes upregulated in granulosa cells while cows with a low AFC presented genes upregulated only in cumulus cells. Taking together with data from other studies, these findings suggest that AFC may influence the molecular network that controls ovarian function ( Ireland *et al*., 2009 ; Mossa *et al*., 2010 ). Considering the importance of communications between oocyte and cumulus cells, as well as the influence of gonadotropins on the cAMP in the two cell types ( Luciano *et al*., 2004 ), it is quite predictable that AFC may really interfere on the rates of embryonic production. However, a precise experiment to consider only AFC and embryo production remains a challenge, since multiple aspects need to be isolated in the experimental design.

As described in Table 1 , the main problem for comparing the AFC data is the classification of animals in groups of high and low AFC. Each team considered a specific strategy. Thus, it is quite difficult to compare the results.

**Table 1:** Results (mean ± SD) from studies comparing *in vitro* embryo production in *Bos taurus*, *Bos indicus* and *Bos indicus-taurus* (crossbred) between high and low AFC groups.

Author	type	AFC	Animals n	PU n	COC’s n	Blastocyst n	Blastocyst rate %
Ireland *et al*., 2007	*Bos taurus*	Low (≤ 15 follicles)	68 ^***^	---	7.5^a^	1.3^a^	29.6
		High (≥ 25 follicles)	37 ^***^	---	29.5^b^	4.9^b^	30.9
Silva-Santos *et al*., 2014	*Bos indicus-taurus*	Low (≤ 10 follicles)	20 ^*^	20	5.8 ± 3.4^A^	0.5 ± 0.8^A^	9.5^A^
		High (≥ 40 follicles)	20 ^*^	20	36.9 ± 13.7^B^	6.1 ± 4.5^B^	16.5^B^
Santos *et al*., 2016	*Bos indicus*	Low (≤ 7 COC’s)	19 ^*^	19	3.8 ± 1.1^α^	0.6 ± 0.6^α^	13.0^α^
		High (≥ 40 COC’s)	22 ^*^	22	40.4 ± 10.6^β^	18.4 ± 6.7^β^	41.9^β^
Monteiro *et al*., 2017	*Bos indicus*	Low (< 15 COC’s)	18 ^**^	216	10.8 ± 0.4	3.6 ± 0.2	33.9
		High (≥ 15 COC’s)	18 ^**^	216	21.2 ± 1.0	7.1 ± 0.4	34.2
Rosa *et al*., 2018	*Bos indicus*	Low (≤ 31 follicles)	356 ^***^	---	536 (total)	203 (total)	38.6
		High (≥ 92 follicles)	356 ^***^	---	617 (total)	251 (total)	40.6

* Animals submitted to OPU only once.

** Animals submitted to OPU more than once.

*** Animals submitted to postmortem ovarian aspiration. ^a,b/A,B/α,β ^For the same author and variable were different (P ≤ 0.05) between the AFCs groups. Adapted from Ireland *et al*. (2007) ; Silva-Santos *et al*. (2014) ; Santos *et al*. (2016) ; Monteiro *et al*. (2017) and Rosa *et al*. (2018) .

Controversial data among studies in AFC classification also are present in both *in vivo* and *in vitro* embryonic production, as shown in Table 2 .

**Table 2:** Results (mean ± SD) from studies comparing *in vivo* embryo production in *Bos taurus* and *Bos indicus-taurus* (crossbred) between high and low AFC groups.

Author	Animals	Number of follicles	Number of flushes	Transferable embryos/ animals
Ireland *et al*., 2007	*Bos taurus*	Low (≤ 15 follicles)	21 ^*^	3.8 ± 0.8^a^
		High (≥ 25 follicles)	19 ^*^	5.4 ± 1.3^b^
Silva-Santos *et al*., 2014	*Bos indicus-taurus*	Low (≤ 10 follicles)	20 ^**^	1.9 ± 2.1^A^
		High (≥ 40 follicles)	20 ^**^	6.9 ± 5.3^B^

* The same female may have been superovulated and collected up to twice.

** One single collection per animal. ^a,b/A,B^For the same author were different (P ≤ 0.05) between the AFCs groups. Adapted from Ireland *et al*. (2007) and Silva-Santos *et al*. (2014) .

Considering data of pregnancy rate and AFC after artificial insemination, it is also interesting to identify the clear differences among the groups, as shown in the Table 3 .

**Table 3:** Reproductive performance of females with high or low follicle count (AFC) after artificial insemination.

**Author**	**Group**	**Pregnancy rate (%)**
**Mossa *et al*., 2012**	Low AFC (≤ 15 follicles)	84
	
	High AFC (≥ 25 follicles)	94
		
**Mendonça *et al*., 2013**	Low (≤ 12 follicles)	51.85
	
	High AFC (≥ 30 follicles)	44.73
		
**Santos *et al*., 2016**	Low AFC (≤ 10 follicles)	58,6
	
	High AFC (≥ 25 folículos)	51,9

Adapted from Mossa *et al*. (2012) ; Mendonça *et al*. (2013) and Santos *et al*. (2016) .

## AFC: consistent data versus new challenges

The strategy used to the classification of females in AFC categories is very different in each author. Each one exhibited a distinct experimental design; some studies did not consider the AFC intermediate group or, for example, the oocytes were recovered in different ways - by OPU or ovaries from a slaughterhouse ( Ireland *et al*., 2008 ; Monteiro *et al*., 2017 ). In some cases, the parameters used to classify females in AFC categories differed considerably among authors ( Morotti *et al*., 2015 ). Therefore, when comparing studies concerning AFC, we should consider how the separation of animals into the categories was made, trying to establish a more balanced comparison.

Moreover, there are differences between the studied subspecies inherent to the physiology of the estrous cycle, which encompass divergences from follicular wave number per cycle to the number of follicles recruited per growth wave ( Baruselli *et al*., 2007 ). For example, Holstein cows tend to present the predominance of two or three waves of follicular growth per estrus cycle ( Ginther *et al*., 1989 ; Wolfenson *et al*., 2004 ), whereas Zebu cows are more related to three to four waves per estrus cycle ( Rhodes *et al*., 1995 ; Nelore - Figueiredo *et al*., 1997 ). Also, *Bos indicus* females recruit more follicles per follicular growth wave than *Bos taurus* females (33,4 ± 3,2 *vs* 25,4 ± 2,5; Carvalho *et al*., 2008 ). Taking those data together, it is easy to realize the challenge to compare studies that present results of distinct subspecies. So, the comparison of AFC with fertility in different breeds should be studied individually.

Furthermore, for a better precision of the factors that interfere with AFC, it is necessary to consider the development phase in which the follicles are aspirated since there is a direct interference on the oocyte competence for IVEP. In this context, Cavalieri *et al*. (2018) , showed that cows submitted to follicular wave synchronization had a better IVEP rate and pregnancy rate after ET when compared to females aspirated on a random day of estrous cycle. Because of that, the strategy for oocyte recovery must be considered when comparing different AFC cows.

In addition to the subspecies, it is also important to consider the influence of the age of the females in the AFC studies, since there are indications, that the ovarian follicular reserve decreases after the female reaches five years, suggesting a decrease in fertility ( Cushman *et al*., 2009 ). The same authors also reported the importance of birth weight as a parameter that influences AFC, but the stage of estrous cycle seems to not interfere in the AFC classification, which facilitates the establishment of pattern (high, intermediate or low AFC) to each cow ( Cushman *et al*., 2009 ).

In summary, according to the literature and data cited above, AFC seems to be correlated with several fertility parameters, and it may be a tool that can contribute to the success of embryo production both *in vivo* and *in vitro*. However, there is a great need to study the real long term impact of AFC on fertility, to establish specific parameters of AFC classification and to understand the physiological causes of the variation in the AFC among individual female cattle.

## Final comments

Despite many studies on antral follicle population, the relationship between AFC, fertility, and efficiency of biotechniques are not fully understood. However, in the context of embryo production, the AFC can be used as an auxiliary tool for the selection of animals with the greater quantitative potential of embryos. Finally, a better understanding of the factors linked to AFC and of the reproductive characteristics from *Bos indicus* and *Bos taurus* may provide adjustments in cattle management and to improve the efficiency of reproductive biotechniques.
